# Supplementary material for: The association between fibroblast growth factor 21 with diabetes retinopathy among type 2 diabetes mellitus patients: a systematic review, meta-analysis, and meta-regression
Source: PeerJ. 2024 Dec 13;12:e18308. doi: 10.7717/peerj.18308 (PMC11648683; doi:10.7717/peerj.18308)
Supplement: Supplemental Information 2 [file peerj-12-18308-s002.docx]

Supplemental Material 2.

**Search strategies**

**MEDLINE** 25 studies

("diabetes retinopathy"[All Fields] OR "diabetic retinopathy"[All Fields] OR (("retinal diseases"[MeSH Terms] OR ("retinal"[All Fields] AND "diseases"[All Fields]) OR "retinal diseases"[All Fields] OR "retinopathies"[All Fields] OR "retinopathy"[All Fields]) AND ("diabete"[All Fields] OR "diabetes mellitus"[MeSH Terms] OR ("diabetes"[All Fields] AND "mellitus"[All Fields]) OR "diabetes mellitus"[All Fields] OR "diabetes"[All Fields] OR "diabetes insipidus"[MeSH Terms] OR ("diabetes"[All Fields] AND "insipidus"[All Fields]) OR "diabetes insipidus"[All Fields] OR "diabetic"[All Fields] OR "diabetics"[All Fields] OR "diabets"[All Fields]))) AND ("fibroblast growth factor 21"[Supplementary Concept] OR "fibroblast growth factor 21"[All Fields] OR ("fibroblast growth factor 21"[Supplementary Concept] OR "fibroblast growth factor 21"[All Fields] OR "fgf 21"[All Fields]))

**Web of Science** 27 studies

1 = ((TS=(diabetes retinopathy)) OR TS=(retinopathy diabetes)) OR TS=(diabetic retinopathy)

2 = (TS=(fibroblast growth factor-21)) OR TS=(fgf-21)

3 = #1 AND #2

**Scopus** 66 studies

( TITLE-ABS-KEY ( "diabetic retinopathy" ) OR TITLE-ABS-KEY ( "diabetes retinopathy" ) ) AND ( TITLE-ABS-KEY ( "fibroblast growth factor-21" ) OR TITLE-ABS-KEY ( "FGF-21" ) )

**Embase** 27 studies
*diabetic retinopathy/ AND exp fibroblast growth factor 21/

**ScienceDirect 103 studies**

"diabetic retinopathy" AND "fibroblast growth factor 21"

**Eligibility**

Reports excluded:

Duplicate (n = 4)

No full-text available (n = 4)

Irrelevant outcomes (n = 8)

Wrong study design (n = 16)

Studies included in review (n = 6)

**Included**

Records identified from primary searching:

MEDLINE (n = 25)

Web of Science (n = 27)

EMBASE (n = 27)

Scopus (n = 66)

ScienceDirect (n = 103)

Duplicate records removed (n = 127)

Potentially relevant articles screened (n = 121)

Records excluded by title and abstract

(n = 83)

Reports assessed for eligibility (n = 38)

**Identification of studies via databases and registers**

**Identification**

**Screening**

Records obtained from previous review (n=0)

Articles recorded in COVIDENCE for initial screening (n = 248)
