# Supplementary material for: The association between fibroblast growth factor 21 with diabetes retinopathy among type 2 diabetes mellitus patients: a systematic review, meta-analysis, and meta-regression
Source: PeerJ. 2024 Dec 13;12:e18308. doi: 10.7717/peerj.18308 (PMC11648683; doi:10.7717/peerj.18308)
Supplement: Supplemental Information 3 [file peerj-12-18308-s003.docx]

**Supplementary Material 3. Raw data**

| **Author, year** | Country | **NDR** | | | | | | **NPDR** | | | | | | **STDR** | | | | | | **NOS** |
| --- | --- | --- | --- | --- | --- | --- | --- | --- | --- | --- | --- | --- | --- | --- | --- | --- | --- | --- | --- | --- |
|  |  | **No** | **Age** | **FGF-21 levels** | **%male** | **BMI** | **HbA1c** | **No** | **Age** | **FGF-21 levels** | **%male** | **BMI** | **HbA1c** | **No** | **Age** | **FGF-21 levels** | **%male** | **BMI** | **HbA1c** |  |
| Esteghamati | Iran | 44 | 55.0 (12) | 103.50 (75.75) | 47.7 | 27.62 | 6.8 (1.77) | 46 | 56.5 (9) | 225.0 (106.0) | 36.9 | 27.97 | 7.65 (1.05) |  |  |  |  |  |  |  |
| Heidari | Iran | 93 | 54.12 (11.27) | 272.00 (27.26) | 34.3 | 27.39 | 7.58 (0.54) | 44 | 56.02 (9.90) | 431.29 (46.30) | 36.4 | 27.22 | 9.13 (0.93) | 47 | 54.96 (10.15) | 361.45 (32.17) | 38.3 | 28.93 | 8.37 |  |
| Jin | China | 345 | 57.11 (11.99) | 326.8 (81.6) | 53.6 | 25.28 | 7.73 (1.88) | 207 | 58.41 (10.13) | 572.7 (79.5) | 51.7 | 25.31 | 8.28 (1.77) | 102 | 58.93 | 625.8 (83.7) | 53.9 | 25.11 (3.74) | 8.49 (1.93) |  |
| Mousavi | Iran | 22 | 54.0 (6.0) | 259.5 | 22.7% | 28.2 | 7.1 (2.0) | 25 | 56.0 (7.0) | 324.0 | 24% | 28.0 | 8.9 (2.6) |  |  |  |  |  |  |  |
| Lin | China | 34 | 59.4 (10.2) | 326.8 (81.6) | N/A | 32.3 | 7.7 (1.2) | 34 | 61.3 (10.1) | 631.9 (73.8) | N/A | 32.7 | 8.1 (0.3) | 49 | 59.2 (12.7) | 669.4 (89.2) | N/A | 33.1 (5.9) | 8.3 (1.7) |  |
| Lee | South Korea | 172 | 60.7 (11.5) | 204 | 50.6 | 26.4 (3.99) | 9.03 (1.82) |  |  |  |  |  |  | 4588 | 62.9 (12.3) | 177 | 58.7 | 26.1 (4.40) | 7.50 (1.30) |  |
|  |  | 710 |  |  |  |  |  | 356 |  |  |  |  |  | 4786 |  |  |  |  |  |  |
